# Supplementary material for: Duplex One-Step RT-qPCR Assays for Simultaneous Detection of Genomic and Subgenomic RNAs of SARS-CoV-2 Variants
Source: Viruses. 2022 May 17;14(5):1066. doi: 10.3390/v14051066 (PMC9143037; doi:10.3390/v14051066)
Supplement: Supplementary file 1 [file viruses-14-01066-s001.zip › Sup/Supplemental Table S1.pdf]

**Supplemental Table S1.** ***gORF1a*** **+** ***sgS*** **duplex RT-qPCR results for RNA extracted longitudinally from the supernatant of TMPRSS2 Vero E6 cells infected with SARS-CoV-2 Washington strain.**

| **Time (Hours)** | ***gORFla* (Ct Mean)** | ***gORF1a* (Ct SD)** | ***gORF1a* (5 x log copies/µg)** | ***sgS* (Ct Mean)** | ***sgS* (Ct SD)** | ***sgS* (5 x log copies/µg)** |
|---|---|---|---|---|---|---|
| 2 | 35.956 | 1.683 | 4.763609023 | 37.549 | | 4.547823597 |
| 4 | 28.326 | 0.065 | 7.058345865 | 29.152 | 0.093 | 6.952462772 |
| 6 | 24.412 | 0.053 | 8.235488722 | 25.528 | 0.019 | 7.990263459 |
| 8 | 23.463 | 0.149 | 8.520902256 | 25.04 | 0.118 | 8.130011455 |
| 12 | 21.34 | 0.048 | 9.159398496 | 23.198 | 0.11 | 8.657502864 |
| 16 | 19.488 | 0.098 | 9.716390977 | 21.772 | 0.025 | 9.065864834 |
| 20 | 18.888 | 0.038 | 9.896842105 | 21.691 | 0.069 | 9.08906071 |
| 24 | 18.888 | 0.012 | 9.896842105 | 22.86 | 0.085 | 8.754295533 |
| 30 | 19.331 | 0.011 | 9.763609023 | 23.454 | 0.066 | 8.58419244 |
| 36 | 20.452 | 0.051 | 9.426466165 | 23.45 | 0.024 | 8.585337915 |
| 42 | 20.293 | 0.075 | 9.474285714 | 24.762 | 0.167 | 8.209621993 |
| 48 | 20.229 | 0.055 | 9.493533835 | 24.743 | 0.222 | 8.215063001 |

*gORF1a* = genomic ORF1a ; *sgS* = subgenomic spike; ; RT-qPCR = real-time reverse transcription PCR; TMPRSS2 = human transmembrane serine protease 2; SARS-CoV-2 = severe acute respiratory syndrome coronavirus 2.
