# Supplementary material for: Duplex One-Step RT-qPCR Assays for Simultaneous Detection of Genomic and Subgenomic RNAs of SARS-CoV-2 Variants
Source: Viruses. 2022 May 17;14(5):1066. doi: 10.3390/v14051066 (PMC9143037; doi:10.3390/v14051066)
Supplement: Supplementary file 1 [file viruses-14-01066-s001.zip › Sup/Supplemental Table S2.pdf]

Supplemental Table S2. ***gORF1a + sgS*** **duplex RT-qPCR results for RNA extracted longitudinally from the supernatant of TMPRSS2 Vero E6 cells infected with SARS-CoV-2, delta variant.**

Time (h)	***gORFla*** **(Ct Mean)**	***gORF1a*** **(Ct SD)**	***gORF1a*** **(5 x log copies/µg)**	***sgS*** **(Ct Mean)**	***sgS*** **(Ct SD)**	***sgS*** **(5 x log copies/µg)**
2	35.223	0.148	4.98406015	-		
4	34.871	0.324	5.089924812	37.945	0.733	4.434421535
6	31.881	0.176	5.989172932	33.095	0.146	5.823310424
8	27.84	0.014	7.204511278	29.08	0.028	6.973081329
12	24.145	0.022	8.315789474	25.899	0.06	7.884020619
16	22.179	0.022	8.907067669	24.264	0.043	8.352233677
20	20.786	0.041	9.326015038	23.189	0.024	8.660080183
24	20.741	0.061	9.339548872	25.979	0.178	7.861111111
30	18.625	0.012	9.97593985	22.508	0.093	8.855097365
36	20.359	0.019	9.45443609	34.77	0.075	5.343642612
42	20.919	0.111	9.286015038	25.196	0.054	8.085337915
48	20.981	0.082	9.267368421	25.087		8.116552119

gORF1a = genomic ORF1a ; *sgS* = subgenomic spike RT-qPCR = real-time reverse transcription PCR; TMPRSS2 = human transmembrane serine protease 2; SARS-CoV-2 = severe acute respiratory syndrome coronavirus 2.
