# Supplementary material for: Duplex One-Step RT-qPCR Assays for Simultaneous Detection of Genomic and Subgenomic RNAs of SARS-CoV-2 Variants
Source: Viruses. 2022 May 17;14(5):1066. doi: 10.3390/v14051066 (PMC9143037; doi:10.3390/v14051066)
Supplement: Supplementary file 1 [file viruses-14-01066-s001.zip › Sup/Supplemental Table S3.pdf]

Supplemental Table S3. ***gORF1a + sgORF3a*** **duplex RT-qPCR results for RNA extracted longitudinally from the supernatant of TMPRSS2 Vero E6 cells infected with SARS-CoV-2 Washington strain.**

Time (h)	*gORFla* (Ct Mean)	*gORF1a* (Ct SD)	*gORF1a* (5 x log copies/µg)	*sgORF3a* (Ct Mean)	*sgORF3a* (Ct SD)	*sgORF3a* (5 x log copies/µg)
2	34.603	1.008	4.87605042	35.496	-	5.407272727
4	27.494	0.314	7.009603842	27.678	0.294	7.776363636
6	23.494	0.071	8.210084034	23.845	0.021	8.937878788
8	22.443	0.07	8.525510204	23.154	0.012	9.147272727
12	20.32	0.033	9.162665066	21.227	0.089	9.731212121
16	18.525	0.093	9.701380552	19.923	0.085	10.12636364
20	17.904	0.02	9.887755102	19.566	0.054	10.23454545
24	17.907	0.03	9.886854742	20.315	0.036	10.00757576
30	18.319	0.004	9.763205282	20.53	0.041	9.942424242
36	19.464	0.068	9.419567827	20.971	0.039	9.808787879
42	19.343	0.053	9.455882353	21.788	0.026	9.561212121
48	19.288	0.045	9.472388956	22.077	0.029	9.473636364

gORF1a = genomic ORF1a ; *sgORF3a* = subgenomic ORF3a; RT-qPCR = real-time reverse transcription PCR; TMPRSS2 = human transmembrane serine protease 2; SARS-CoV-2 = severe acute respiratory syndrome coronavirus 2.
