# Supplementary material for: Duplex One-Step RT-qPCR Assays for Simultaneous Detection of Genomic and Subgenomic RNAs of SARS-CoV-2 Variants
Source: Viruses. 2022 May 17;14(5):1066. doi: 10.3390/v14051066 (PMC9143037; doi:10.3390/v14051066)
Supplement: Supplementary file 1 [file viruses-14-01066-s001.zip › Sup/Supplemental Table S4.pdf]

Supplemental Table S4. ***gORF1a + sgORF3a*** **duplex RT-qPCR results for RNA extracted longitudinally from the supernatant of TMPRSS2 Vero E6 cells infected with SARS-CoV-2, delta variant.**

**Time (h)**	***gORFla*** **(Ct Mean)**	***gORF1a*** **(Ct SD)**	***gORF1a*** **(5 x log copies/µg)**	***sgORF3a*** **(Ct Mean)**	***sgORF3a*** **(Ct SD)**	***sgORF3a*** **(5 x log copies/µg)**
2	34.023	0.43	5.050120048	37.462	-	4.811515152
4	33.349	0.576	5.25240096	36.91	1.613	4.978787879
6	30.779	0.403	6.023709484	31.627	0.539	6.57969697
8	26.773	0.094	7.225990396	27.517	0.102	7.825151515
12	23.041	0.068	8.346038415	24.264	0.049	8.810909091
16	21.128	0.018	8.920168067	22.713	0.067	9.280909091
20	19.796	0.053	9.319927971	21.446	0.025	9.664848485
24	19.6	0.164	9.378751501	22.894	0.206	9.226060606
30	17.611	0.065	9.975690276	20.2	0.111	10.04242424
36	19.352	0	9.453181273	23.457	0.029	9.055454545
42	19.833	0.027	9.308823529	22.614	0.019	9.310909091
48	20.792	1.396	9.021008403	22.742	0.022	9.272121212

gORF1a = genomic ORF1a ; *sgORF3a* = subgenomic ORF3a; RT-qPCR = real-time reverse transcription PCR; TMPRSS2 = human transmembrane serine protease 2; SARS-CoV-2 = severe acute respiratory syndrome coronavirus 2.
