# Supplementary figures and images for: Duplex One-Step RT-qPCR Assays for Simultaneous Detection of Genomic and Subgenomic RNAs of SARS-CoV-2 Variants
Source: Viruses. 2022 May 17;14(5):1066. doi: 10.3390/v14051066 (PMC9143037; doi:10.3390/v14051066)

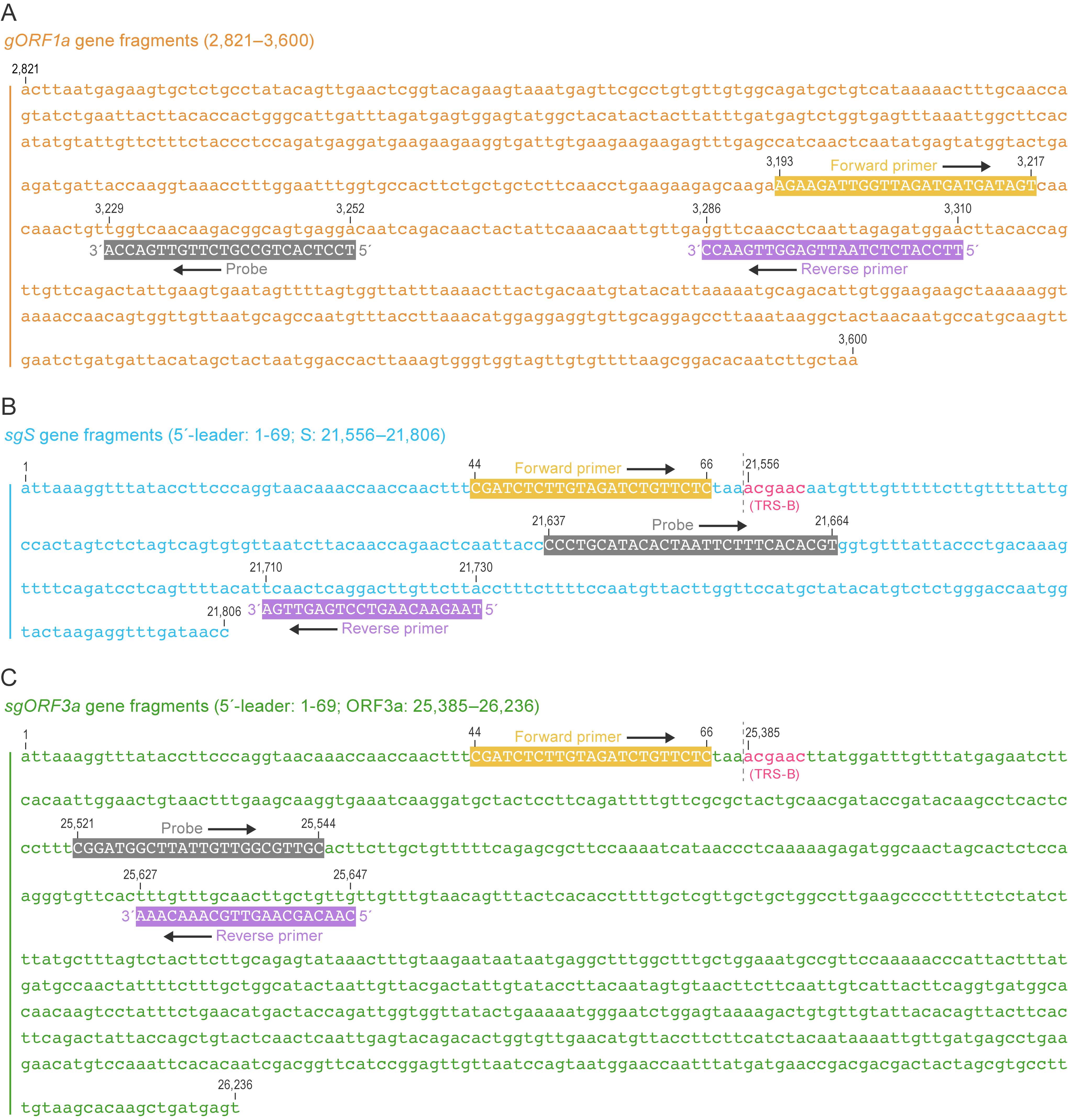

Supplement: Supplementary file 1 [file viruses-14-01066-s001.zip › Sup/Sup Figure S1.tif]

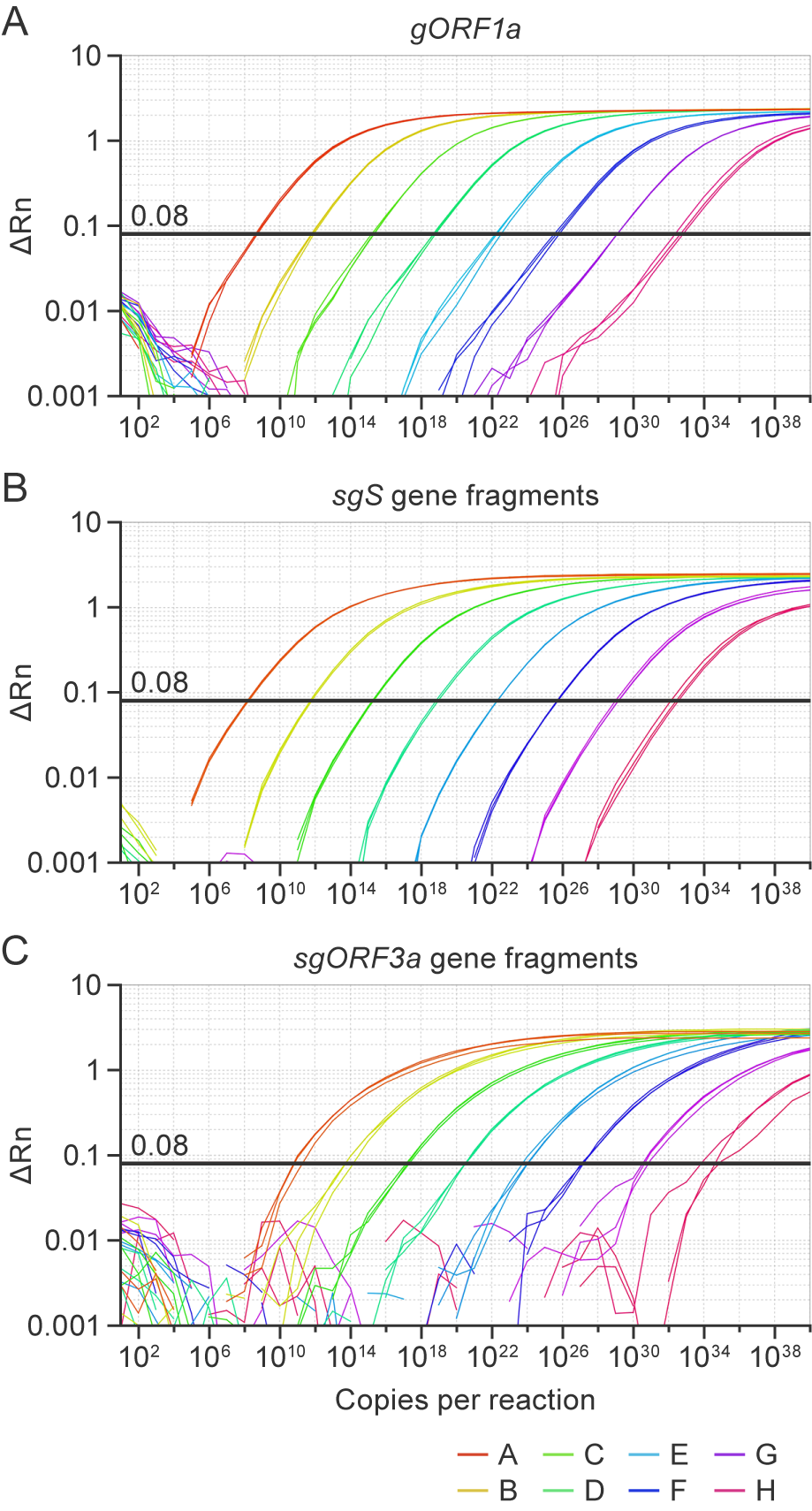

Supplement: Supplementary file 1 [file viruses-14-01066-s001.zip › Sup/Sup Figure S2.tif]

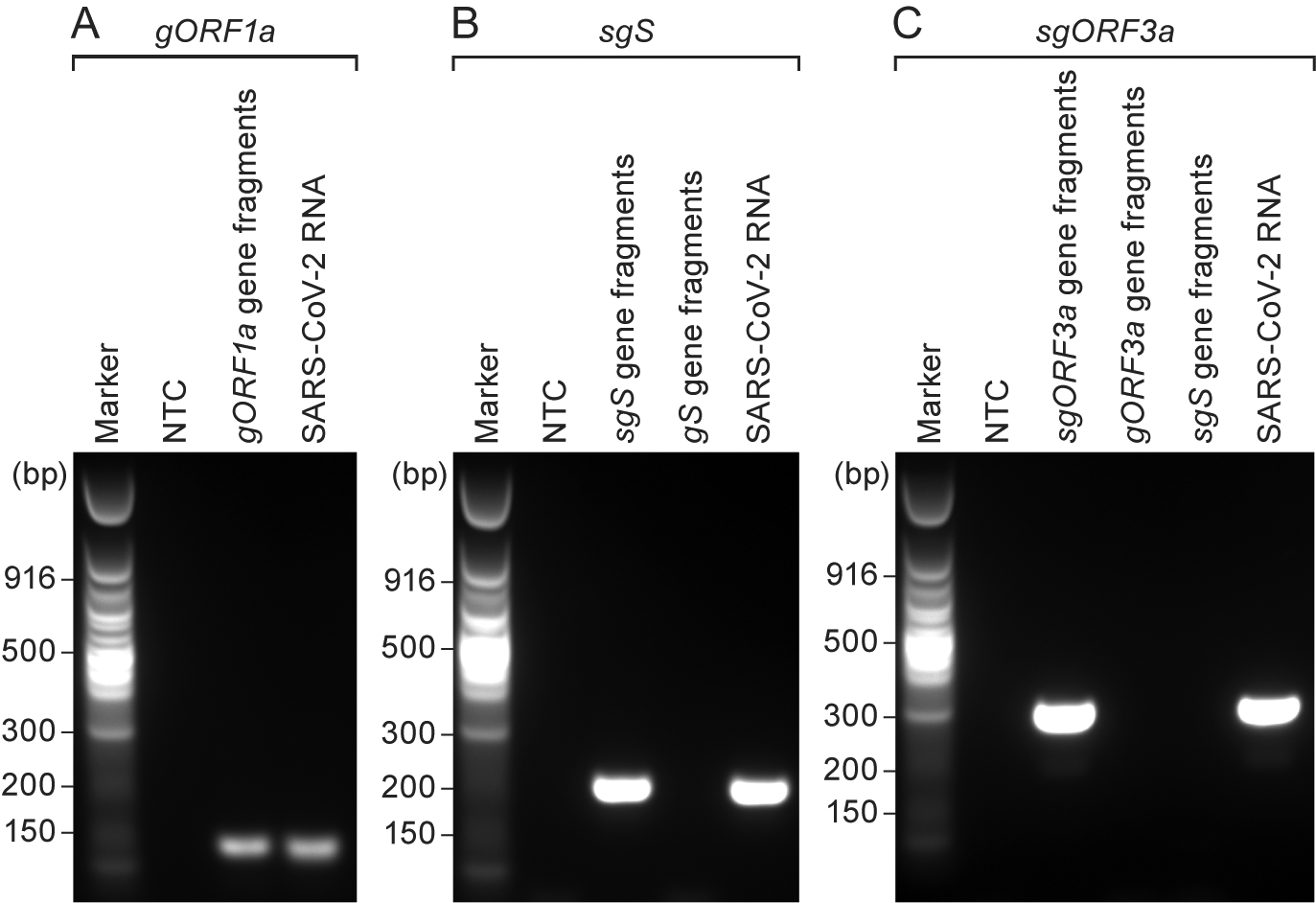

Supplement: Supplementary file 1 [file viruses-14-01066-s001.zip › Sup/Sup Figure S3.tif]

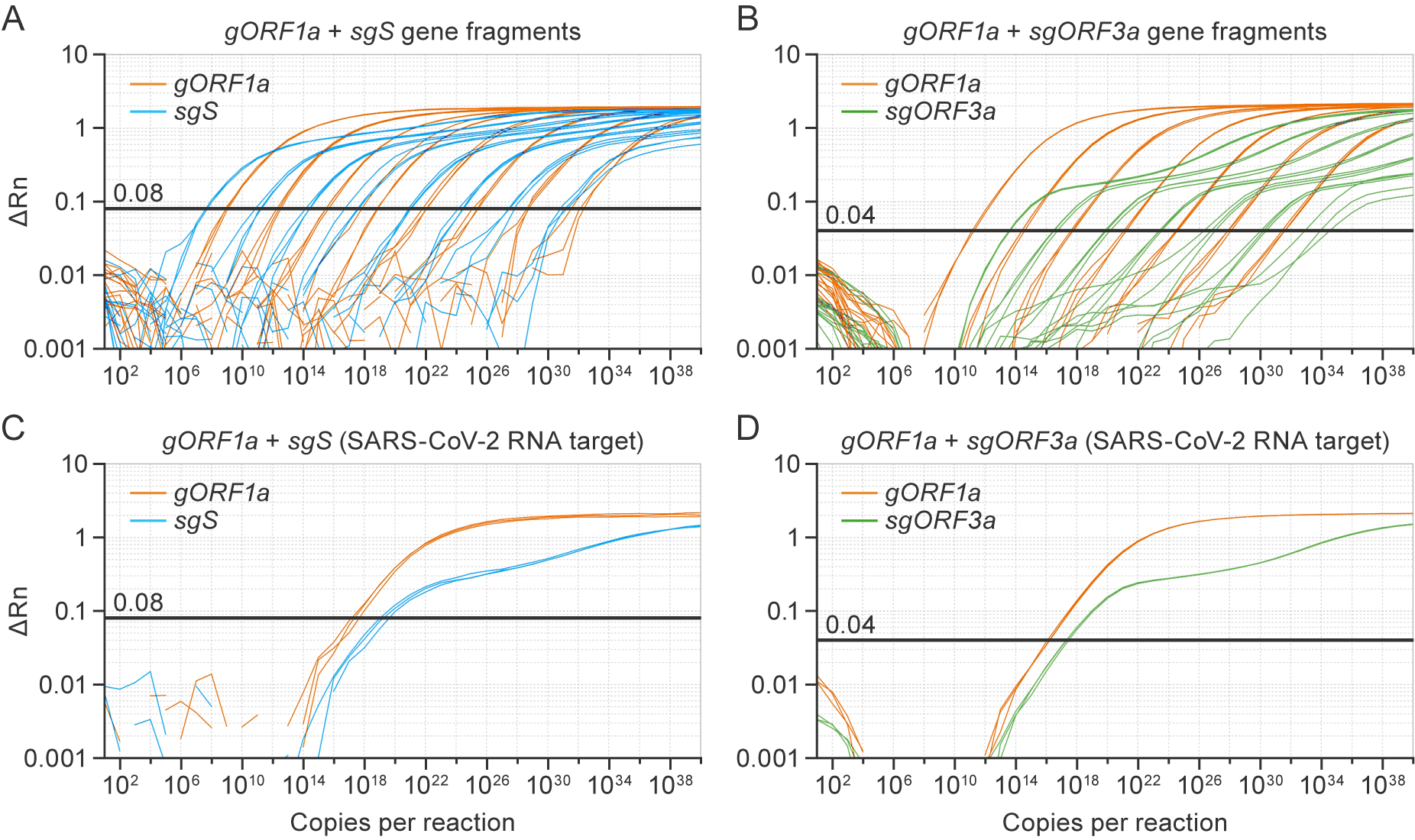

Supplement: Supplementary file 1 [file viruses-14-01066-s001.zip › Sup/Sup Figure S4.tif]

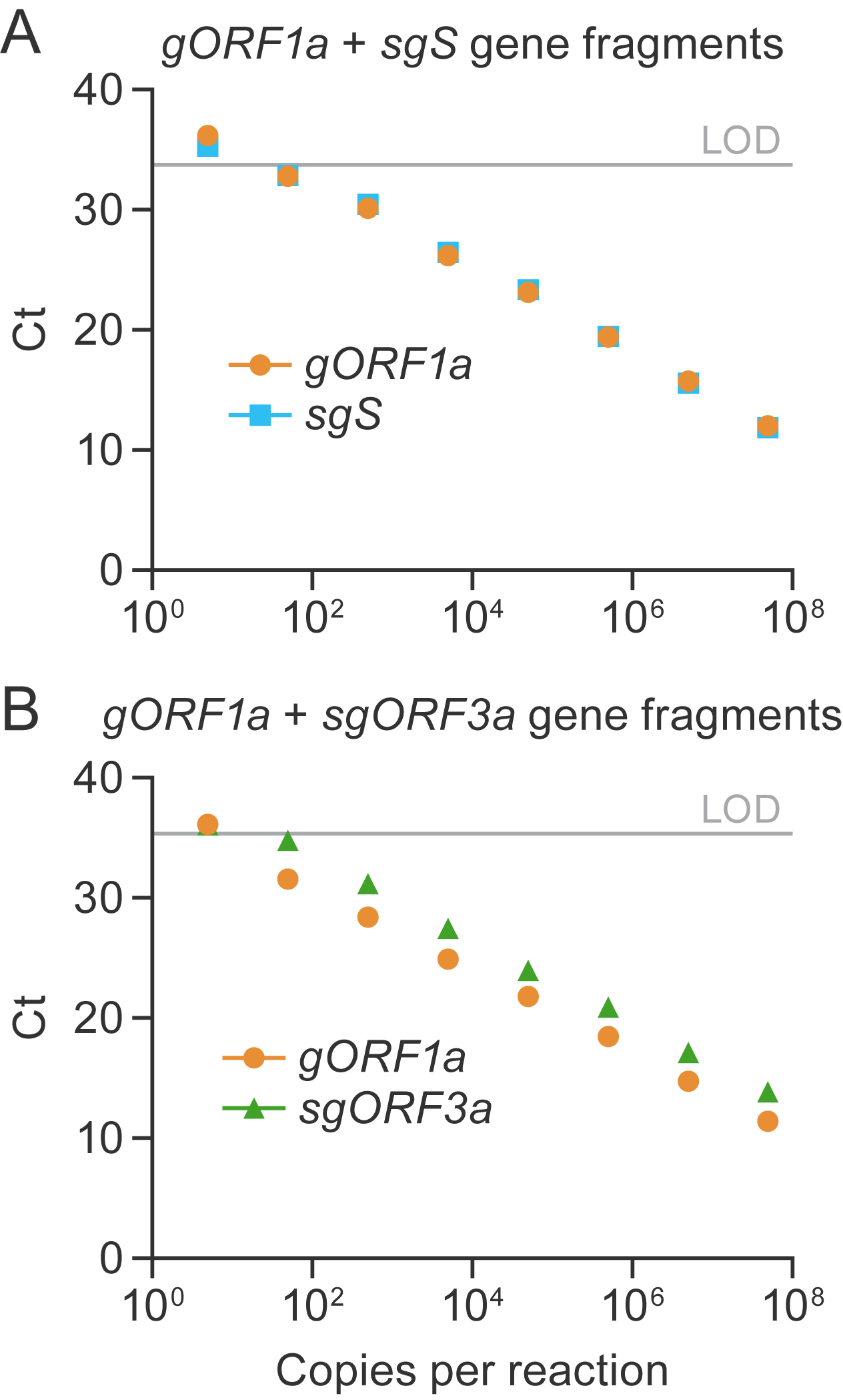

Supplement: Supplementary file 1 [file viruses-14-01066-s001.zip › Sup/Sup Figure S5.tif]

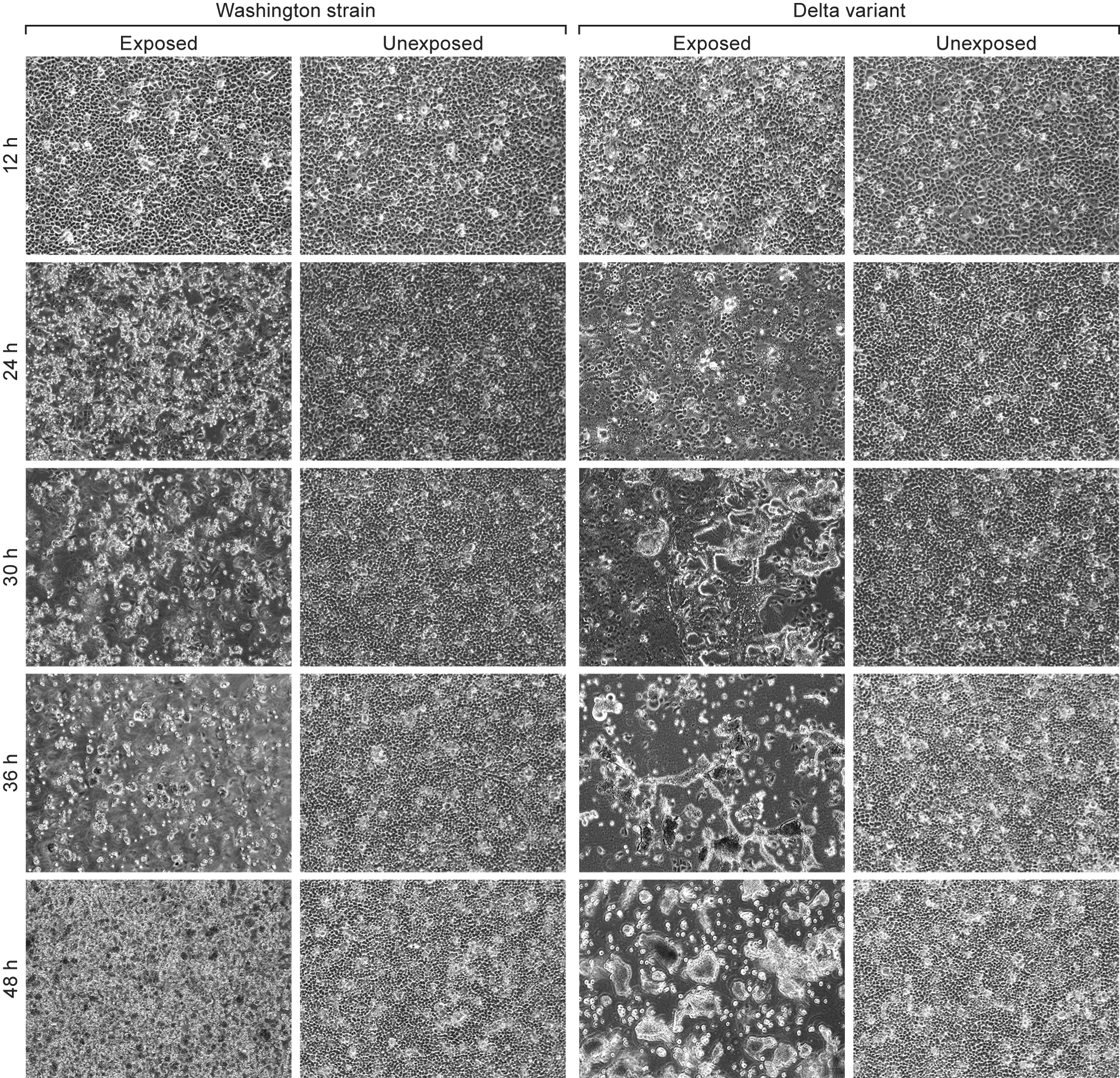

Supplement: Supplementary file 1 [file viruses-14-01066-s001.zip › Sup/Sup Figure S6.tif]
